# Axillary Artery Injury and By-Pass Restoration After Open Reduction of a Chronic Shoulder Dislocation: A Case Report

**DOI:** 10.7759/cureus.23594

**Published:** 2022-03-29

**Authors:** Athanasios Patousis, Nikolaos P Sachinis, Christos Yiannakopoulos, Panagiotis Givissis

**Affiliations:** 1 Orthopaedics and Trauma, "Georgios Papanikolaou" General Hospital, Thessaloniki, GRC; 2 Orthopaedic, IASO Hospital, Athens, GRC; 3 School of Physical Education and Sports Science, National and Kapodistrian University of Athens, Athens, GRC; 4 School of Medicine, Aristotle University of Thessaloniki, Thessaloniki, GRC

**Keywords:** open reduction, dislocation, chronic, shoulder, axillary artery

## Abstract

Injury of the axillary artery after open reduction of a chronic shoulder dislocation is a rare and life-threatening condition. We present a case of an elderly woman suffering from a chronic shoulder dislocation which was addressed initially with close reduction and secondarily, after re-dislocation, with open reduction. Intraoperatively axillary artery rupture was established. By-pass restoration with a saphenous vein graft successfully managed the complication. The humeral head was immobilized in the glenoid with temporary K-wires. A CT-angiography was performed on the first and second days postoperatively.

## Introduction

Injury of the axillary artery after chronic shoulder dislocation addressed with open reduction is rare. Few cases in English publications have been reported that demonstrate injuries of the vessel following dislocation of the humeral head [1,2]. This report studies the case of an elderly woman with a history of chronic shoulder dislocation, the intraoperative open reduction, the management of the arterial rupture, as well as the final clinical outcome. This case highlights the need for careful intraoperative dissection and protection of vital structures and reduction forces control in patients with chronic shoulder dislocation, and the backup of a vascular surgeon.

## Case presentation

**Figure 1:**
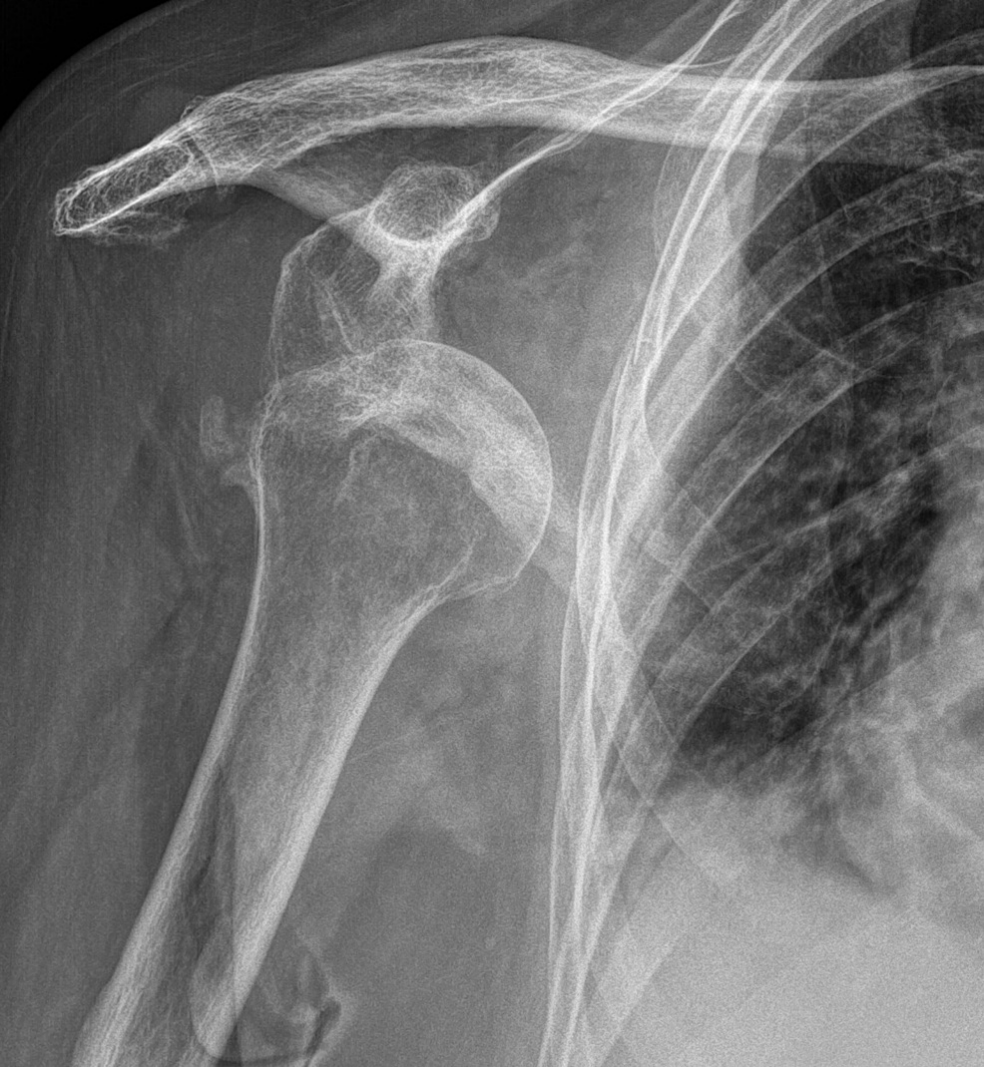
Initial anteroposterior radiograph of the right shoulder revealing an anterior glenohumeral dislocation at A&E Department.

A 75-year-old woman with BMI of 29 and no medical history suffered an anterior dislocation of the right shoulder when falling onto her out-stretched upper extremity. Approximately, six weeks after that injury, the patient visited the Accident and Emergency (A&E) department of our institution; the reasons for not seeking medical attention sooner were unknown. The patient denied having pain when the injured arm was rested in adduction and internal rotation with the elbow flexed at 90° degrees, but could not abduct the arm since the injury. In the primary examination, the patient appeared to have normal neurological and vascular status. Radiographs of the right shoulder confirmed an anterior dislocation of the glenohumeral joint (Figure 1). The patient had an otherwise undistinguished medical history. Following admission to the hospital, reduction under sedation in the operating room was attempted. After seven efforts, using the Hippocratic method, the humeral head was reportedly seen in place during intraoperative radiologic imaging with a C-arm. Neurovascular status was intact following the final attempt. The patient was discharged 24 hours later, and a follow-up was booked seven days post-reduction.

**Figure 2:**
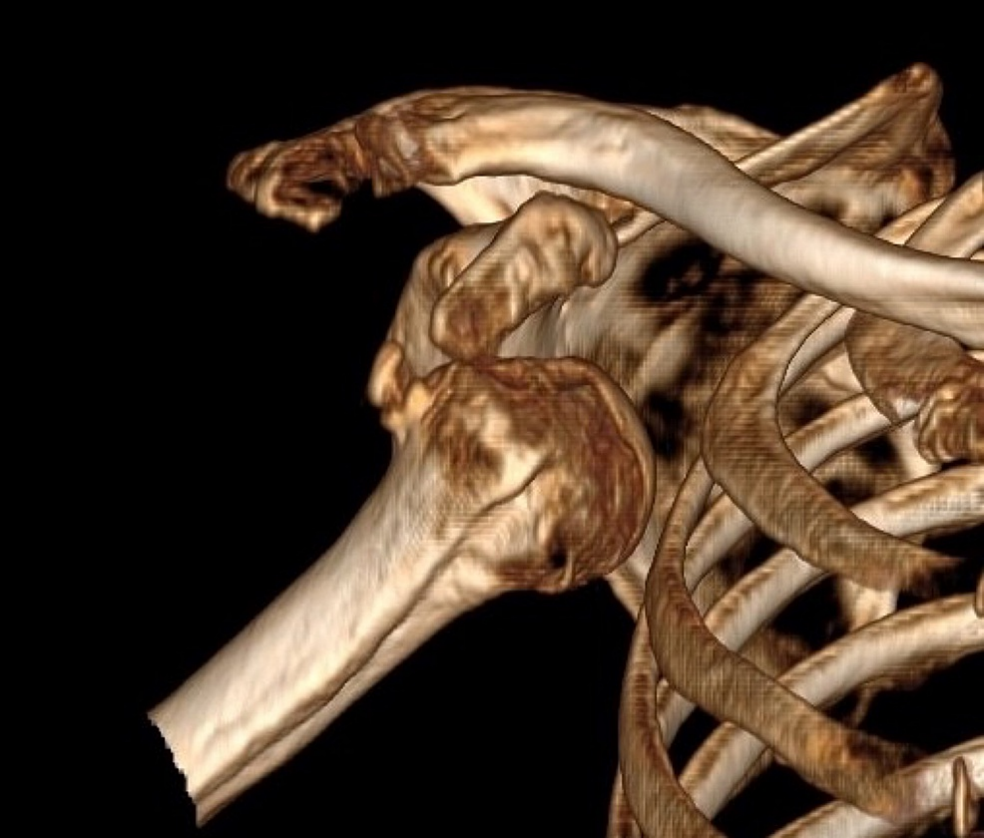
3D CT scan of the left shoulder revealing a Hill-Sacks lesion and the anterior humeral dislocation with no fracture.

**Figure 3:**
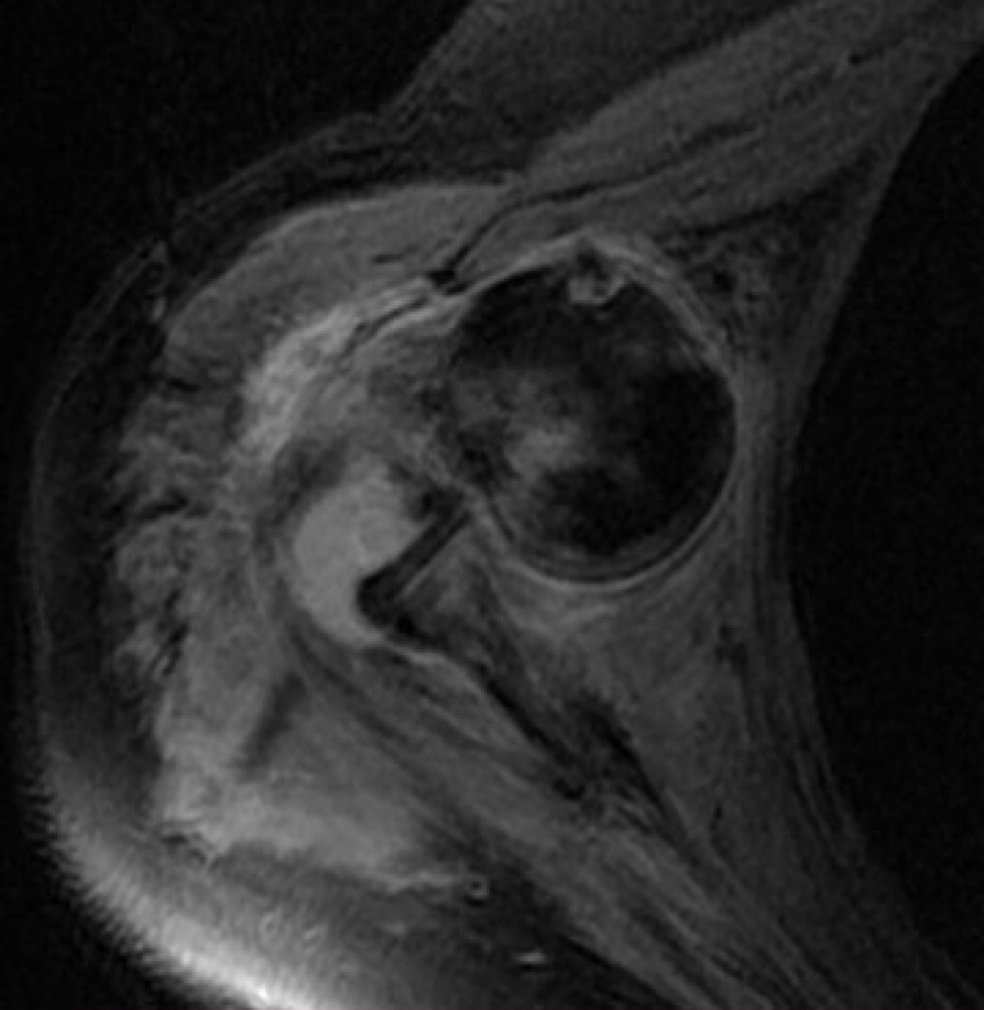
MRI STIR imaging showing a Bankart lesion and posterolateral edema of humeral head. The rotator cuff was intact. STIR: Short Tau Inversion Recovery

During the patient’s follow-up, the shoulder was found re-dislocated, despite the shoulder being immobilized with a specific shoulder brace. No neurovascular deficits were ascertained. The patient was advised to have a computed tomography (CT) scan and magnetic resonance imaging (MRI) of the shoulder. The patient denied admission at the time and agreed to have these examinations only as an outpatient. Two weeks passed until the patient was seen again at the hospital’s shoulder clinic. The CT scan did not detect any fracture, but a Hill sacks defect was present on the posterosuperior part of the humeral head, extending in less than 25% of the head’s diameter (Figure 2). The MRI indicated neither rotator cuff tear nor cartilage lesions. A Bankart type lesion was found in the anteroinferior part of the labrum (Figure 3). The patient was informed about the risks and benefits of possible surgical options and agreed to an open Bankart repair and capsular shift. She refused being operated on immediately and revisited the shoulder clinic after two weeks for admission (10 weeks after the initial injury; four weeks after the closed attempt of shoulder reduction). The patient’s relatives were informed in regards to the shoulder injury and treatment options and were also queried about the reasons for the postponement of her treatment. The patient was reportedly generally reluctant in visiting doctors and hospitals, especially during the coronavirus disease 2019 (COVID-19) pandemic.

**Figure 4:**
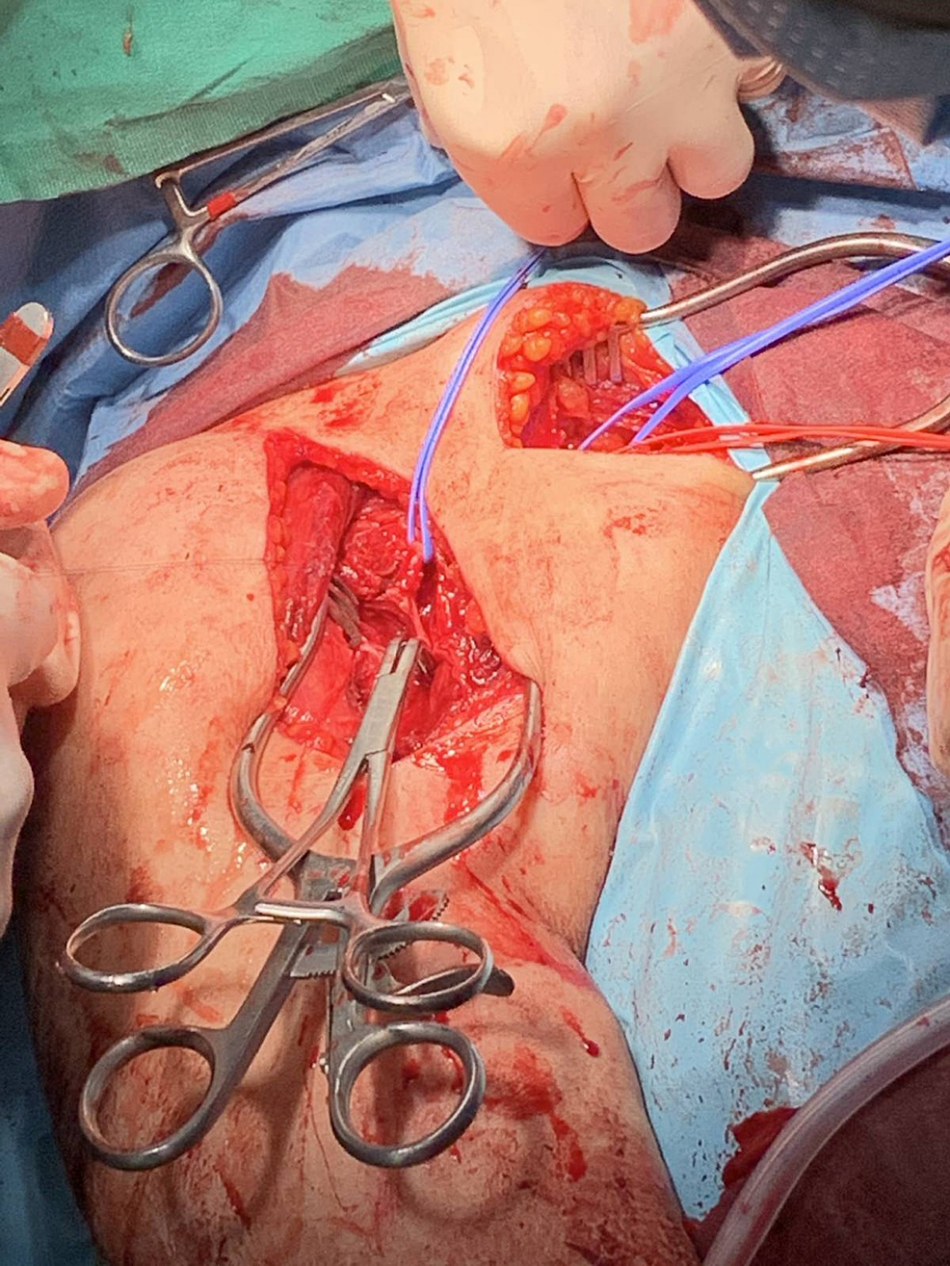
Intraoperative photograph of the patient’s right shoulder. Another proximal approach can be seen that is utilized for recognition of the subscapular artery and clamping during restoration of the axillary artery with by-pass.

After admission and preoperative assessment, the patient was brought to the operating room within 24 hours. Under general anesthesia and interscalene block, a deltopectoral approach was performed. The subscapularis was split and reduction of the dislocated humeral head was attempted. During manipulation for reduction, it was noted that bleeding was coming from behind the conjoined tendon. Attempts for finding the bleeding vessel were initially unsuccessful due to the deep location of the vessel and the rapid increase in bleeding. Vascular surgeons were called immediately and pressure was applied with compress gauzes to control the bleeding until the arrival of those surgeons. Radial and brachial pulses were absent. Upon arrival of the vascular surgeons, the artery was temporarily ligated centrally under the clavicle by a second approach (Figure 4). Osteotomy of the coracoid was performed by the vascular surgeons in order to explore the axillary artery injury.

**Figure 5:**
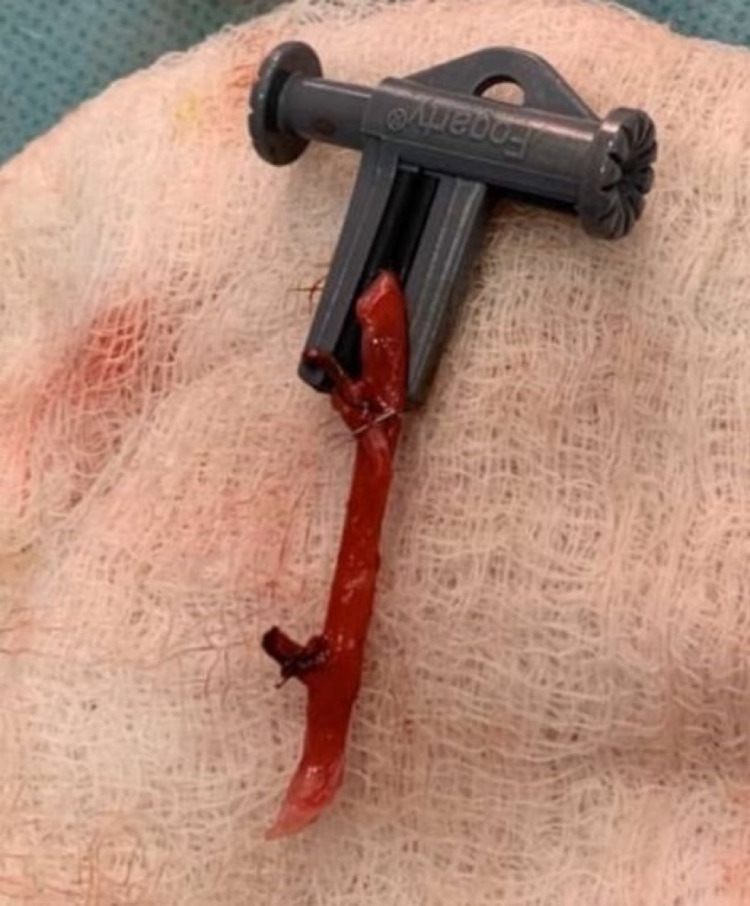
Saphenous vein graft from the right leg that was utilized for by-pass restoration of the artery.

**Figure 6:**
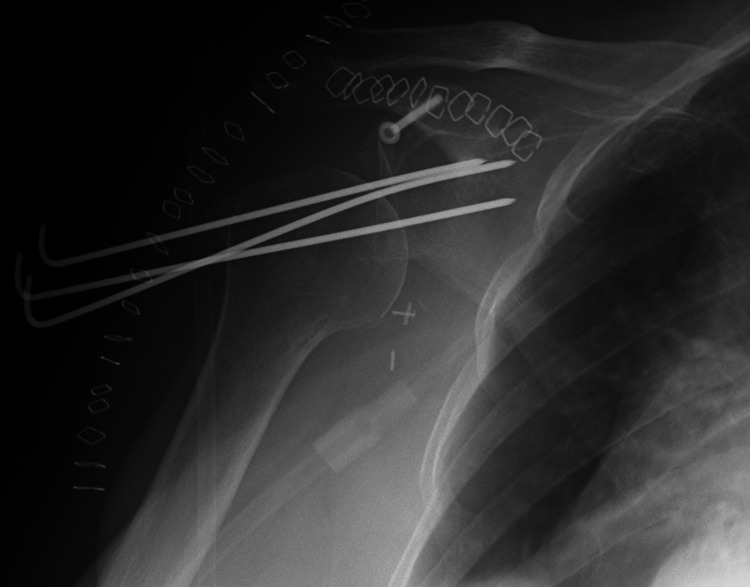
Immediate postoperative radiograph showing temporary immobilization of the humeral head with 3 k-wires into the glenohumeral joint and screw fixation of the coracoid in its anatomic position.

The artery was found torn at its third segment and, due to its attenuation, primary closure was not feasible. As a result, a saphenous vein graft from the right leg was utilized for the by-pass restoration of the artery (Figure 5). The humeral head was partially reduced and additional attempts of reduction and labrum repair/shift were not performed, as no retractors could be placed without staying away from the artery and further traction could jeopardize the vascular repair. Then, immobilization of the humeral head in the accomplished partially subluxated position was performed with three Kirschner wires (K-W) from the humerus to the glenohumeral joint, to protect the by-pass restoration from immediate postoperative dislocation (Figure 6).

**Figure 7:**
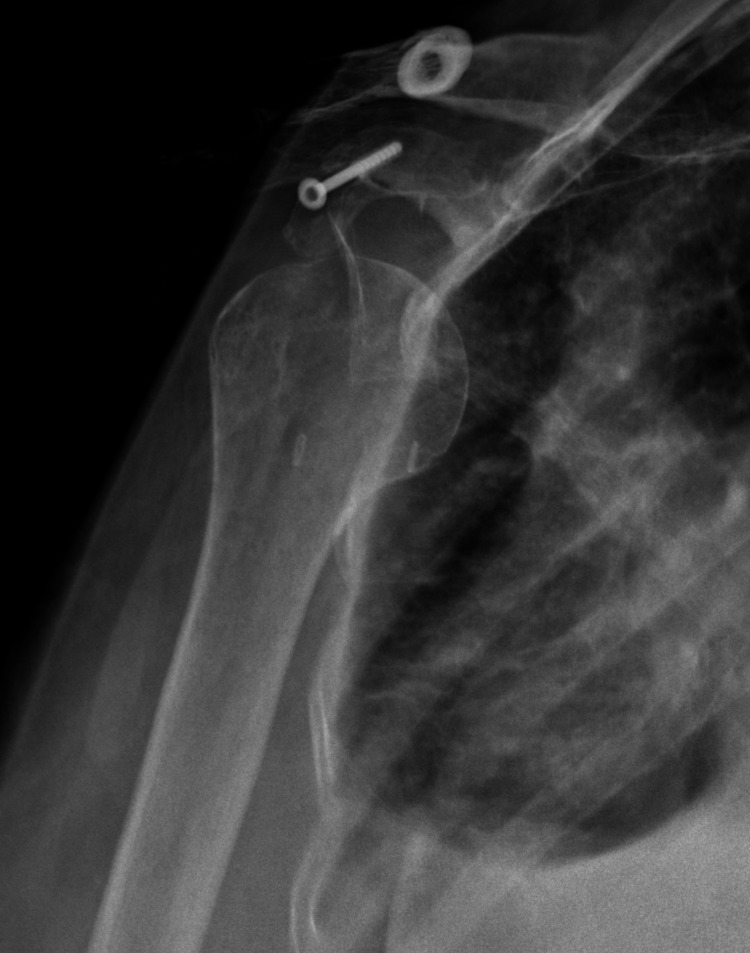
Three-month postoperative anteroposterior radiograph revealing anterior shoulder dislocation.

**Figure 8:**
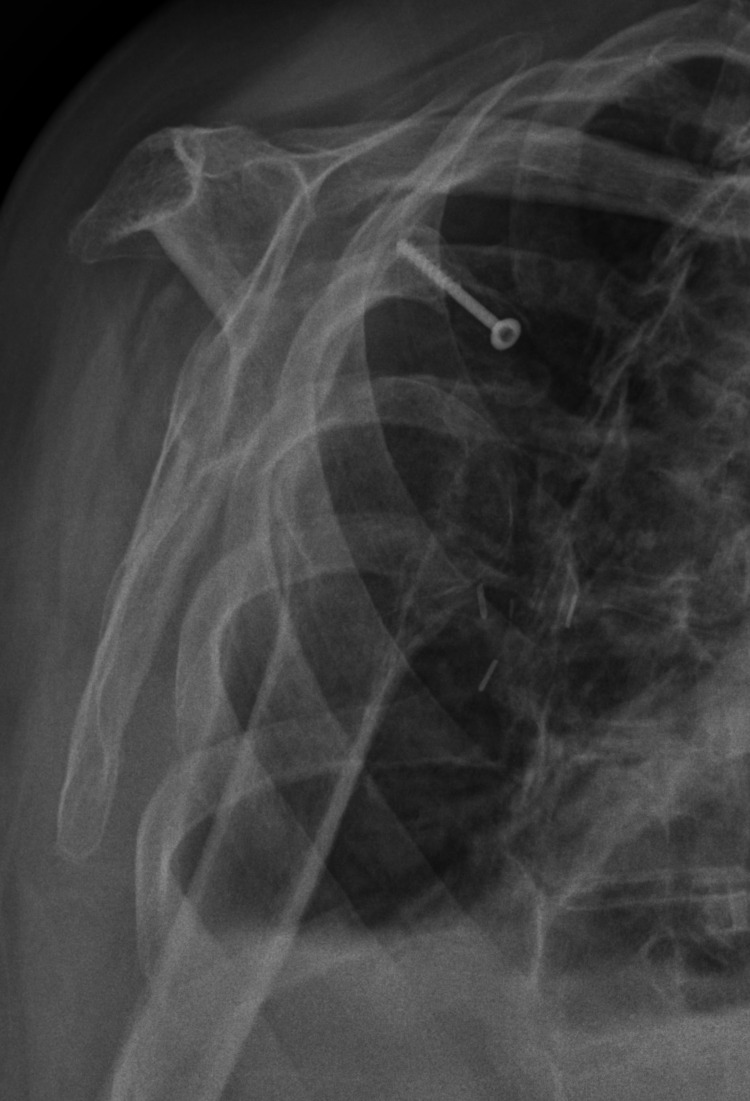
Three-month postoperative Y-view radiograph revealing anterior shoulder dislocation.

Finally, the coracoid was fixated in its anatomic position with a partially threaded 4 mm screw. Bilateral radial and brachial pulses were present equally. The wound was closed and the arm was placed in a shoulder abduction brace. Neurological postoperative clinical examination revealed radial and musculocutaneous neuropraxia which resolved completely within four weeks. After the procedure, anticoagulation therapy with a regimen of low molecular weight heparin (enoxaparin 4000 units/daily) was used and sustained for about four weeks. Following the previous therapy, aspirin dose of 100 mg/day was introduced as an antiplatelet therapy. Two CT-angiographies were performed on the first and third days postoperatively with no vascular deficits. The patient was seen clinically and radiologically one week, three weeks, six weeks, twelve weeks, and six months postoperatively. The K-W were removed at four weeks. The humeral head was found dislocated at the three-month follow-up; the patient reported no pain, but extremely limited shoulder movements (Figures 7-8). A reverse shoulder arthroplasty was scheduled at the final follow-up to restore the functionality of the shoulder; thus, dependence on the rotator cuff after months of attenuation and inactivity would be redundant. Unfortunately, the patient passed away due to COVID-19 complications before reverse shoulder arthroplasty was performed.

## Discussion

Injury of the axillary artery is uncommon after anterior shoulder dislocation because it lies deep within the soft tissues of that region. Although there are a few cases in the literature related to axillary artery injury following anterior shoulder dislocation, there is only one after open reduction. Yamazaki et al. described a similar case after open reduction and axillary artery injury which was addressed with percutaneous transluminal angioplasty with a one-year follow-up [3]. Trauma to the artery may happen due to penetration or blunt force [4]. Mortality and morbidity rates range from 3 to 33% [4]. Mortality is higher in patients who have suffered penetrating trauma whereas morbidity is higher in patients who have suffered blunt trauma [4]. Injury of the axillary artery is found and needs to be addressed in approximately 1% of all glenohumeral dislocations and is less common than injury of the popliteal artery after a knee dislocation [1]. Even though extreme forces are used during reduction, and have been accused of such vessel injuries, similar trauma may happen when less force is used, e.g., for reduction of recurrent dislocations [5].

The pectoralis minor muscle separates the axillary artery into three parts [5]. The third part has the most common incidence of rupture or dissection following a blunt trauma [6,7]. According to current knowledge, anatomically, the third segment of the axillary artery is less mobile due to its outcoming circumflex humeral arterial branches. This anatomic detail makes it far more susceptible to a traction injury. The patient in this case also sustained injury to the third segment of the vessel. There are many types of injuries, such as dissection, lesions, internal avulsion, bruises, thrombosis, and aneurysms that can cause damage to the axillary artery [7].

The main mechanisms concerning the traction injury of this reported case include i) abrupt torsion of the artery above pectoralis minor, ii) glenohumeral joint arthritis and recurrent shoulder dislocations that formed fibrotic scar tissue that enclosed the artery, and iii) atherosclerosis that diminished the tensibility of the artery causing it to be prone to damage by traction and/or compressive forces during reduction of the humeral head [2,8,9]. Pathophysiologically, a break of the intimal first layer while the adventitia remained intact caused an intimal flap and a subintimal hematoma. Further traction tore the artery at that injured site. In this case, the artery was most likely further attenuated, and the laceration became wider when increased pressure was applied to the site to stop the bleeding.

A careful neurovascular examination is recommended preoperatively in all patients who suffer from anterior shoulder dislocation, especially the elderly ones. Axillary artery dissection may even occur after partial rupture of the vessel that results in severe upper-limb ischemia. These factors require vigilant vascular examination in elderly patients. The patients who have symptoms of suspected injury of the axillary artery, such as pain, numbness, cold ipsilateral hand (when compared to the other), as well as muscle weakness, sensitivity, or stiffness after shoulder dislocation need CT- angiography regardless of the presence of pulses [3]. Additionally, brachial plexus injury with permanent neurological deficits may be caused by hematoma or aneurism of the axillary artery. CT-angiography may be done as a routine in chronic dislocation cases to prevent fatal complications and establish the artery’s route and proximity with the dislocated humeral head. It is of major importance that damage to the axillary artery is promptly detected to prevent bleeding and succeed in revascularization. In such cases, the gold standard to determine the type and the expansion of the arterial injury and the appropriate surgical approach is CT-angiography. Management of these injuries involves a variety of treatment options, such as percutaneous transluminal angioplasty, arterial suture repair, and axillobrachial or extra-anatomic bypass grafting [3].

## Conclusions

In conclusion, although rare, axillary artery rupture during open reduction of a chronic anterior shoulder dislocation is a limb-threatening incident, which however can be successfully restored; in the present case with a saphenous vein by-pass grafting. We highlight the need for meticulous preoperative neurovascular examination in elderly patients with chronic shoulder dislocation. Surgical planning with CT angiography and cooperation with vascular surgeons may be warranted in such cases, in order to avoid possibly devastating complications.
